# Fast Three-Dimensional Posture Reconstruction of Motorcyclists Using OpenPose and a Custom MATLAB Script

**DOI:** 10.3390/s23177415

**Published:** 2023-08-25

**Authors:** Emmanuele Barberi, Massimiliano Chillemi, Filippo Cucinotta, Felice Sfravara

**Affiliations:** Department of Engineering, University of Messina, 98166 Messina, Italy; massimiliano.chillemi@unime.it (M.C.); ficucinotta@unime.it (F.C.); fsfravara@unime.it (F.S.)

**Keywords:** OpenPose, 3D human posture, motorsport, posture assessment, ergonomics, computer vision, digital twin

## Abstract

Ergonomics focuses on the analysis of the interaction between human beings and their working environment. During the riding of a motorbike, ergonomics studies the rider’s posture on the motorbike. An incorrect posture can lead to physical and psychological discomfort, and can affect the perception of risk and the handling of the motorcycle. It is important for motorcyclists to adopt a good riding posture, for their health and road safety. The aim of this work is to propose a fast, cheap, and sufficiently robust method for the 3D reconstruction of the posture assumed by a motorcyclist. The stereo vision and the application of OpenPose made it possible to obtain a 3D reconstruction of the key points, and their evolution over time. The evaluation of the distances between the 3D key points, which represent the length of the various parts of the body, appears to remain sufficiently stable over time, and faithful to the real distances, as taken on the motorcyclist themself. The 3D reconstruction obtained can be applied in different fields: ergonomics, motorsport training, dynamics, and fluid dynamics analysis.

## 1. Introduction

### 1.1. Context

Ergonomics is the discipline that studies the interactions between human beings and the environment in which we operate [1]. In the specific case of motorcycle riding, ergonomics focuses on the analysis of the posture that the rider assumes on the bike.

It is important to consider that an incorrect posture can adversely affect the health of the rider. For example, the compression of the spinal nerve caused by an incorrect position can lead to pain in the neck, back, and shoulders [2]. In addition, a poor posture can also affect the driving stability, increasing the risk of falls. A bad posture can also cause discomfort to the user (physical and psychological), and can affect the perception of risk, and the ability to maneuver. Therefore, assuming a good driving posture is not only important for the health of the rider, but also for road safety. The results of an ergonomics study can be useful in characterizing the posture of the rider, and improving the design of the motorcycle. An ergonomics study can be very useful in increasing the safety, comfort, and performance of the rider and a possible passenger [3]. It is therefore evident the need to obtain a design based also on ergonomics and, therefore, on the rider’s comfort. In order to achieve these results, all of the stakeholders need an efficient and fast method of evaluating the posture of the rider in several situations. 

### 1.2. Literature Review

Several works were conducted in this field, mostly based on the assessment of the ergonomic angles of the human body using virtual manikins (2D or 3D) [4]. Barone and Lo Iacono [5] proposed a method to assess the comfort felt on a motorbike, by considering both the static posture and the dynamic behavior, by means of a simulated driving test. In the work of Eida Nadirah et al. [6], the comfort level of two new bike seats was evaluated, in order to identify the best option between the two configurations. A sample of 100 subjects was taken, to acquire the anthropometric data from which the two seats were designed. Afterwards, the participants tested the seats and, through a questionnaire, their level of comfort was evaluated. Tony et al. [2] evaluated the intensity of muscle strain in different positions, through the use of both objective and subjective measurements. In the work, rapid upper limb assessment (RULA) [7] analysis was used to identify the optimal posture to minimize muscle strain while riding a motorcycle, and to prevent musculoskeletal injury. As well as RULA, another analysis, called rapid entire body assessment (REBA) [8], exists. In a similar way to RULA, REBA associates a score with each ergonomic angle, to quantify the risk of injury. In the study by Arunachalam et al. [4], a dataset of the anthropometric characteristics of a user class for the ergonomic design of motorcycles was created. 

Based on previous studies, it is noted that a knowledge of human posture that is as true to reality as possible is essential to good ergonomic design. A faster approach to the problem is to use computer vision and artificial intelligence (AI) techniques. In particular, OpenPose [9] is an open-source software. It can also detect key points of the human body in real time, using computer vision and deep learning techniques. This software can be used to monitor human movement, and to analyze posture in real time, without the use of markers or wearable sensors. The system is based on a convolutional neural network (CNN) trained on a vast dataset of annotated images depicting people in different postures. The software works by detecting key points of the human body, such as joints and extremities. OpenPose is used in a wide range of applications, including biomechanics, posture analysis, physiotherapy, gestures, safety, virtual reality, and sports. The method combines the location information of the identified key points with an object recognition method, to detect dangerous driving behaviors. In the work by Carputo et al. [10], the movement of a virtual motorcycle rider is studied for the determination of the center of gravity (CoG). This parameter, indeed, plays a fundamental role in the simulation of vehicle dynamics, and in the design phase of the motorcycle, as the rider and motorcycle cannot be considered as separate systems. Chen et al. [11] studied falling behavior among the elderly, considering the standing-up mechanism of the human body after a fall. Park et al. [12] developed a real-time push-up counter that can distinguish those performed correctly from those performed incorrectly. Jafarzadeh et al. [13] analyzed, in real time, the key points for a hurdles athlete. The work also shows the OpenPose limitations with an acquisition using a single camera when the person framed is in profile. Yadav et al. [14] developed a yoga posture recognition system using a hybrid deep learning (DL) model based on CNN and long short-term memory (LSTM). In the work of Thar et al. [15], a method for the self-learning of certain yoga postures is proposed. The comparison of the position performed by the user is compared to a reference that is considered correct. The algorithm returns a skeleton that assumes a color in relation to how much the assumed pose differs from the correct one. Lin et al. [16] present a posture evaluation system, focusing on two fitness exercises. Using a recurrent neural network (RNN), the system analyzes the positions of the joints in each frame of a video of a user performing a fitness movement, and evaluates the quality of the movement, in order to correct any wrong movements that could produce injuries. In the work by Lin et al. [17], OpenPose is used to monitor students in the classroom. Essentially, as shown in the aforementioned works, the software analyzes 2D images captured using a single camera, and returns information about the x–y coordinates of the key points detected. It is also possible to obtain the third spatial coordinate, z. This has been widely proven in several studies found in the literature that use different techniques to obtain the coordinates of the key points of the human body in three dimensions. By means of these techniques, it is possible to obtain a three-dimensional reconstruction of the human posture. In Nakano et al. [18], a three-dimensional reconstruction of key points is obtained, using five cameras. The accuracy of the reconstruction is compared with the 3D representation obtained with a marker-based system. Pagnon et al. [19], through the use of a multiple camera system to obtain the 3D skeleton, proposed a framework to build a bridge between OpenPose and OpenSim [20], an open-source software with which it is possible to perform a dynamic simulation of the human body. In the work of Huang and Nguyen [21], the 3D key points of the skeleton are obtained with three Kinect sensors. Labuguen et al. [22] compare the performance of a 3D marker-less body reconstruction (with four cameras) with a marker-based system. The paper of Kim et al. [23] shows a comparison between the OpenPose and Kinect systems, taking as the reference a golden standard system, such as Xsens. The comparison shows that both systems are reliable when it comes to marker-less 3D reconstruction, but OpenPose achieves a higher level of accuracy, and encounters fewer issues related to possible occlusions. The authors, in the present work, have dealt with a topic that is rarely discussed in the literature: the 3D marker-less reconstruction of the posture of a motorcycle rider in real time, to be applied in a context of ergonomics, intended as a design to assure motorbike riders’ wellness. The current state of the art in this field primarily relies on 2D acquisition systems to investigate the posture of motorcycle riders. Arunachalam et al. [4] presented a review paper on motorcycle-riding posture, emphasizing the importance of accurately assessing the rider posture to enhance comfort, and reduce discomfort-related incidents. The authors explain that the main methods used for posture assessment involve the 2D acquisition of joint angles from a lateral view. Another interesting paper by Stolle et al. [24] employs a camera placed at the rear of the bike to evaluate the position of the rider’s back, using OpenPose and Canny edge detection. Once again, this approach is limited to 2D. Arunachalam et al. [25] demonstrate the significance of capturing markers of the rider from different views, and utilize a 2D approach to assess various parameters. The primary objective of this work is to overcome the limitations of the 2D approach, and explore the use of OpenPose in conjunction with an algorithm implemented in MATLAB, to reconstruct a 3D scene of the rider. This enables a comprehensive assessment of posture, through the incorporation of all the key characteristics. Another interesting method of 3D reconstruction could be the use of the depth cameras widely used in the rehabilitation sector. Avola et al. [26] proposed this type of device for the implementation of a rehabilitation framework based on a 3D virtual environment. Another interesting paper is proposed by Lafayette et al. [27]; in this paper, the authors analyze several devices that use the RGB-D technology in the biomechanical field. Other authors [28] have tried to improve the global registration provided by depth map cameras, using feature matching and refinement steps. The use of depth cameras forces the potential end-user to use a particular device; the idea of this application is to use the minimum quantity of simple cameras that anyone could have, without having to buy a particular device. In addition, the authors have experience with the use of OpenPose and 3D reconstruction from a previous study [29], where a machine learning (ML) algorithm was implemented for the self-evaluation of the performance of a gymnastic exercise (the static squat). This work is based exclusively on the 2D reconstruction of key points from multiple images taken at different angles. In the motorbike field, 3D reconstruction is still essential to obtaining good results, because there is the necessity to know anatomic angles in three dimensions. The main novelty of the paper is the application of OpenPose in a non-standard context; a context where the proper orientation of cameras is crucial to capturing all the necessary key points in each frame. The findings aim to provide a solid starting point for anyone interested in using 3D reconstruction methods in a field such as motorcycling, where the current standard for posture assessment is 2D images.

### 1.3. Aim of the Paper and Outline 

The aim of the paper is to propose a method based on OpenPose and MATLAB algorithms, with which it is possible, quickly and cheaply, to obtain a 3D reconstruction of a motorbike rider’s posture, by means of key points. This posture can be used to assess the ergonomic angles of the human body on different types of motorcycle. The main difference with respect to the other works found in the literature is that there is the possibility to obtain real-time posture assessments. In fact, the user can position themself on the bike, appropriately fixed on the ground, trying different postures. It is reasonable to think that this method could have multiple applications:

- Posture assessment with ergonomic angle evaluation
- The dynamic analysis of the human body with the use of OpenSim software
- The interaction of the body with the environment (fluid–dynamic, for example)
- Postural indication during training, to maximize safety, comfort, and even sports performance

The paper starts with a description of the materials and methods used; in particular, the generic workflow followed during the experimental tests, the experimental setup for the OpenPose reconstruction, the camera calibration, and the 3D reconstruction of the key points. This part could be very useful to readers interested in implementing the same procedure. The second part of the paper describes the results of the 3D acquisition and, in general, the main aspect of this particular application. A discussion chapter will be proposed regarding the main considerations about the results obtained and, finally, the conclusion will summarize the main findings, the main weaknesses, and future developments. 

In general, this paper serves as a starting point for the implementation of a standard procedure to evaluate the posture of a person riding a road bike. The primary contribution of this work is to suggest and validate an experimental setup for the 3D reconstruction of the posture of a person riding a road bike. In fact, the literature review reveals that this topic has not yet been adequately addressed, except in a peripheral manner. Typically, the evaluation of a bike in terms of comfort and posture is conducted via general online applications, with a simplified 2D image ([30,31]). This can lead to the overlooking of certain characteristics that can only be assessed through a 3D reconstruction. In order to overcome all of these limitations, the aim of this research is to propose a new approach that takes advantage of OpenPose capabilities, and the features of MATLAB. The end-users of the procedure proposed could comprise not only riders, but also manufactures and researchers. The final 3D reconstruction of the rider could be used as a boundary condition in several different other studies; for example, rigging and skinning for the reconstruction of a digital twin of the human body on a bike, a virtual reality application for bike customers, or the reconstruction of a skeleton with muscles and ligaments for a dynamic evaluation of the forces acting on the human body. 

## 2. Materials and Methods

### 2.1. Workflow

**Figure 1:**
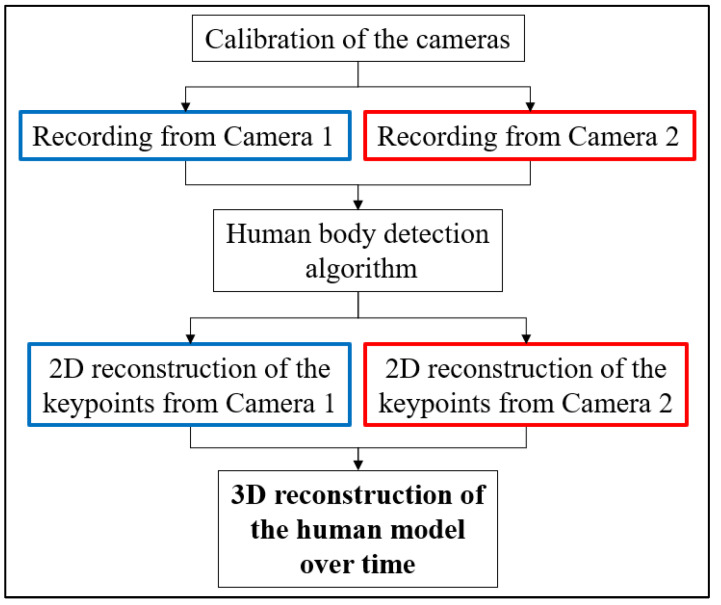
Flowchart of the work.

Figure 1 shows the flowchart of the work conducted. Two fixed cameras are used to record the scene (a rider on a road motorbike). It is fundamental to have a minimum of two different cameras, in order to obtain the videos from two different points of view for the 3D reconstruction of the scene. Of course, increasing the number of cameras used during the acquisition could lead an increase in the capacity to acquire 3D key points, but it also increases the costs and the complexity of the experimental setup. In this study, the approach is to simplify this procedure, in order to achieve a very lean experimental setup. The video from each camera is the input for the pose estimator (OpenPose), from which the coordinates of the key points (in json format) of the human body in two dimensions are obtained. In order to reconstruct the scene in a 3D environment, the cameras have been calibrated via a special board; this procedure is fundamental to obtaining the intrinsic and extrinsic parameters of the two cameras, and the orientation of one camera with respect to the other one. In this way, the two cameras are uniquely located in the three-dimensional space, and it is possible to perform the triangulation of the key points. Knowing the 3D key points allows us to obtain the posture of the rider on the motorcycle. It is fundamental to a correct triangulation process that each key point is simultaneously present in the two videos; this entails, as reported later, great difficulties in positioning the cameras. 

### 2.2. Experimental Setup and OpenPose Application 

The subject of investigation is a rider on a road motorbike. The experimental setup used for the acquisition of videos consists of two fixed identical webcams (see Table 1 for the technical features).

**Figure 2:**
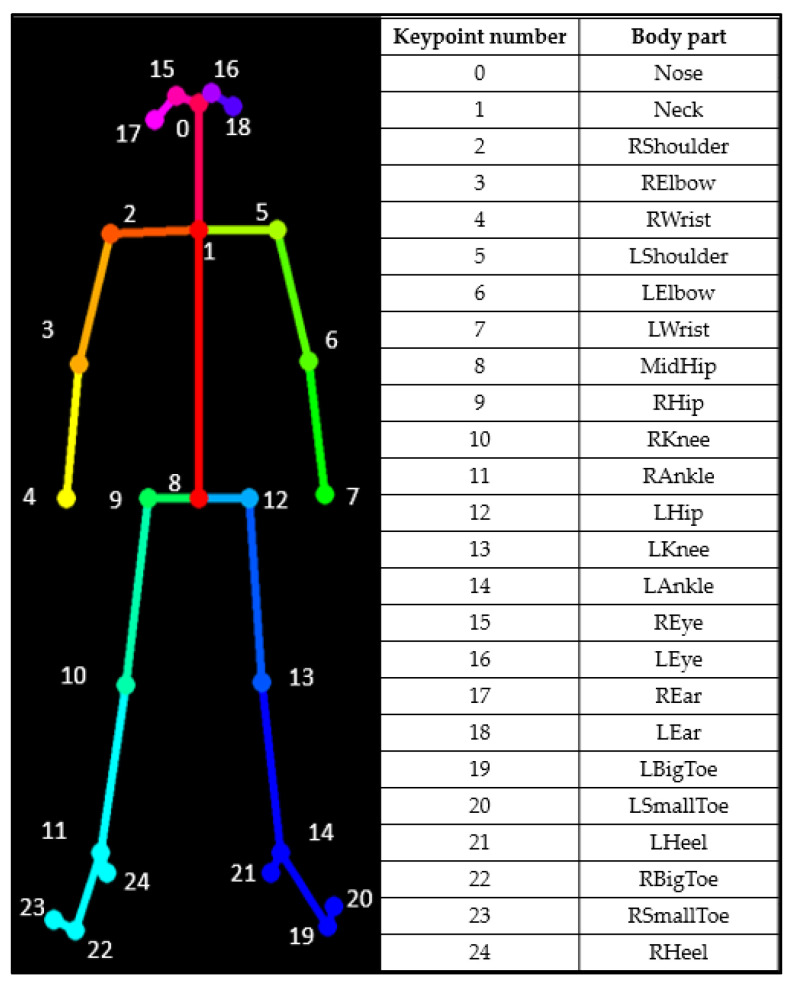
The key points detected via OpenPose.

Videos from the two cameras are processed in real time via OpenPose, which identifies 25 points of the human body, as shown in Figure 2. The outputs of this phase are json files containing the x–y coordinates of the detected points. OpenPose does this for every frame in the videos. 

**Figure 3:**
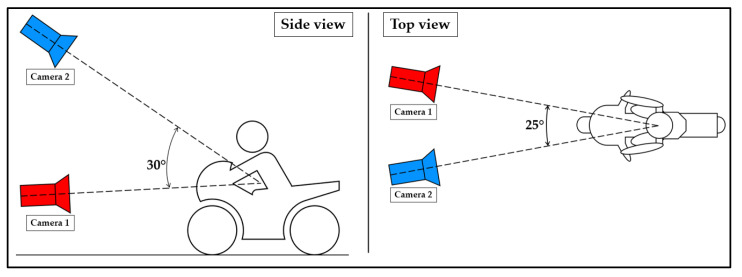
Experimental setup schematization.

Several experiments have been conducted in order to identify the best position for the two cameras. The main goal of this phase is to identify the best position for the cameras, to capture in both videos the whole body of the rider and, therefore, ensure that all the 25 key points are simultaneously captured via OpenPose. The final setup involves the two cameras having a relative angle of 30° in the side-view plane, and 25° in the top-view plane, as shown in Figure 3. 

**Figure 4:**
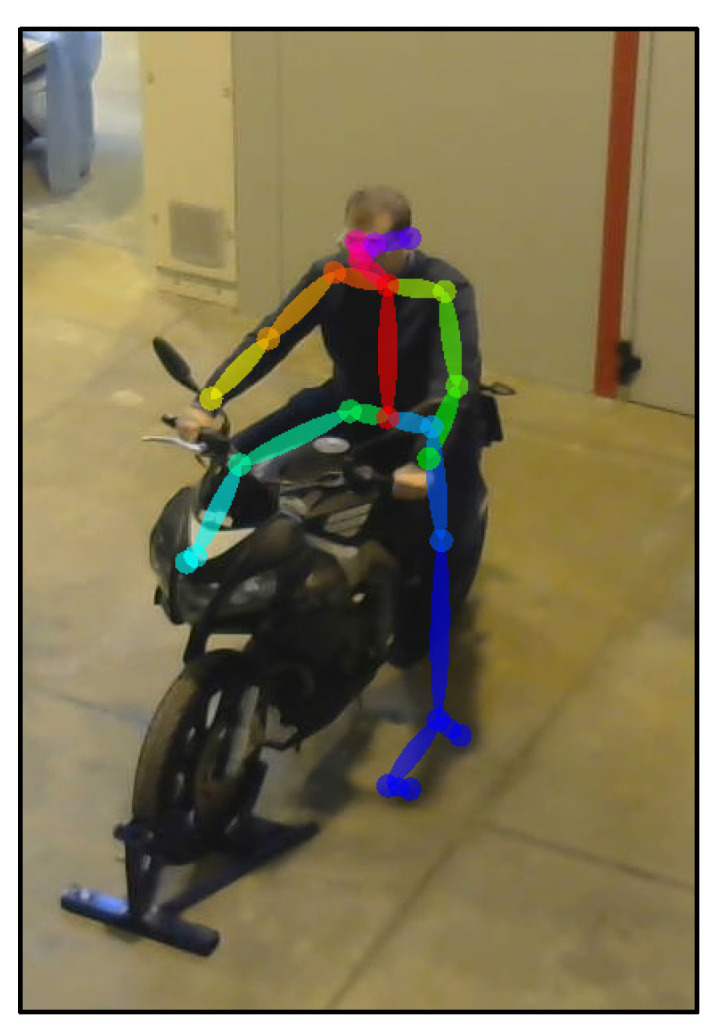
An instance of failed reconstruction due to bad lighting.

The angles obtained are the maximum ones, guaranteeing the full efficiency of the method. The authors’ initial idea was to use a stereo camera that was higher positioned than the other one. However, the test was unsuccessful, as raising the camera from the frontal acquisition position made it more challenging for OpenPose to detect the key points. In general, the best position suggested for the detection of the key points and the subsequent 3D reconstruction would be a 0–90° configuration but, for this kind of application, this configuration was not applicable, because only part of the body could be captured. The first difficulty in this type of setup is the presence of an obstacle, such as the bike, which does not allow for the use of excessively high stereo angles. In fact, larger angles than the ones proposed lead to the erroneous identification of key points, or even the missing of the key points belonging to that part of the body (the only way to accept this configuration was to state that the body was symmetrical). This is because they would prevent the simultaneous acquisition of key points in the same frame. As mentioned above, another important problem that emerged during the acquisition and assessment of the key points captured via OpenPose was acquisition with Camera 2 positioned at a high-value angle in the side view. With the increase in this angle, the algorithm of OpenPose has difficult identifying the features of the body, probably because it is strongly orientated toward the detection of key points in images captured in front or lateral view (the ML algorithm of OpenPose has been trained with images with people captured principally in front or lateral view). The proposed configuration is the best among all the tested ones, and can serve as a reference point for other researchers in the field. As an example of these problems, in Figure 4, it is possible to see the problem relative to the presence of shadows, and that the right lower leg is entirely covered by the front fairing, with this particular configuration of the cameras. 

In order to use only a pair of webcams for the experiment, the authors have identified the best configuration among all the tested setups in the paper. This configuration allows all the key points to be kept simultaneously present in all the frames. 

### 2.3. Camera Calibration

**Figure 5:**
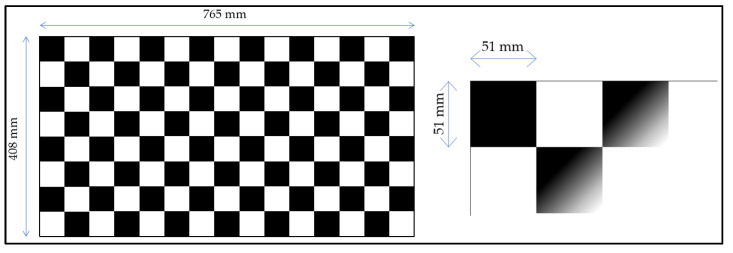
The chessboard used for camera calibration, and its main dimensions.

For 3D reconstruction, it is necessary to uniquely identify the location in the space of the two cameras. This was done through the use of a calibration board. Once the cameras were fixed, a video was recorded, in which the calibration board was present. Figure 5 shows the main dimensions of the calibration board (408 mm × 765 mm). The number of rows and columns is, respectively, 8 and 15 and the single square measures 51 mm × 51 mm. 

During the video capture, the board was moved by means of translation and rotation for each axis (x, y, and z), paying attention to it always being clearly visible in both shots.

**Figure 6:**
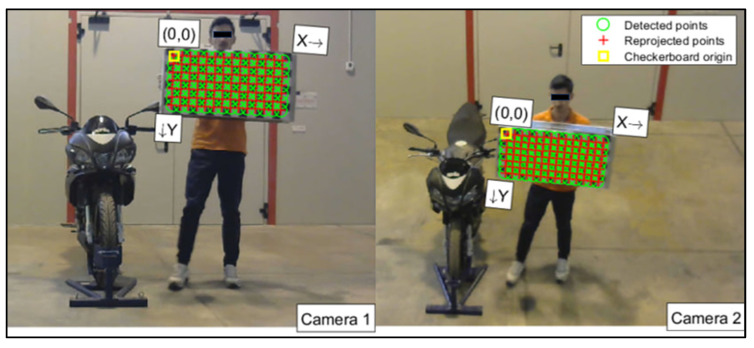
Chessboard detection in the camera calibration process.

**Figure 7:**
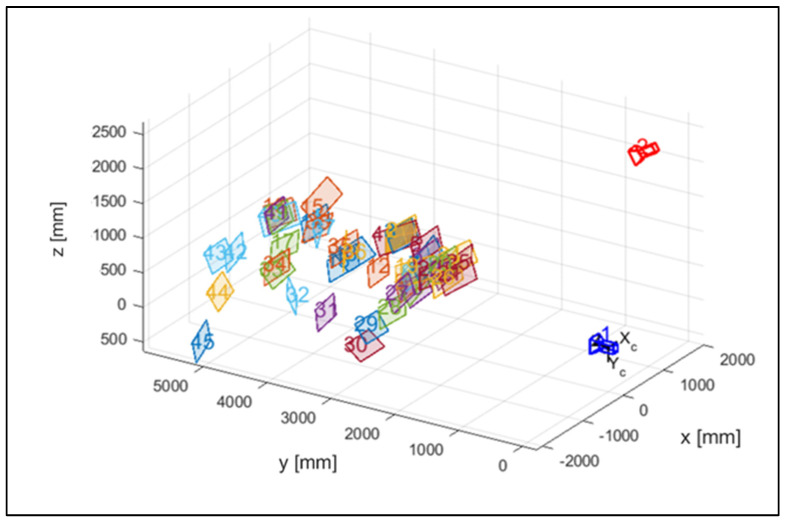
The estimated positions of the calibration board and cameras.

From the two videos, synchronized in post-processing, all the frames were extracted, because the camera calibration needed pairs of pictures. The calibration procedure was performed using the Stereo Camera Calibrator app of MATLAB [32]. This phase allows the calibration of two cameras, to estimate the depth of objects in the scene. The calibration takes place via the recording of images of a known pattern (chessboard), which are used to estimate the intrinsic and extrinsic parameters of the cameras themselves. In this way, it is possible to correct the distortions of the images, and obtain the matrices of rotation and translation that allow the transformation of the coordinates of the 3D points present in the scene from the reference system of one camera to that of the other. Figure 6 shows the chessboard detection procedure, and the calibration process. For a perfect calibration, the red crosses must be inside the green circles. The axis system shown in Figure 6 is only a local reference system for the calibration board. It does not coincide with the reference system of the reconstruction (Figure 7).

In Figure 7, the different positions of the checkerboard during the calibration phase are represented in a tridimensional domain with the two cameras.

**Figure 8:**
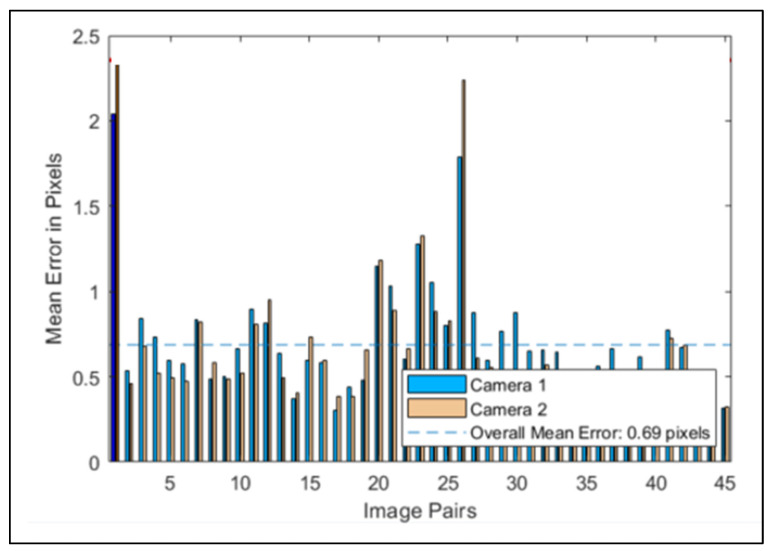
The reprojection errors, calculated via the Stereo Calibrator app.

In Figure 8, the mean reprojection errors are plotted. The reprojection errors provide a qualitative measure of accuracy: a reprojection error is the distance between a key point detected on the checkerboard in the calibration image, and the corresponding world point projected onto the same image. Different pairs of photos have different errors, and the overall mean error, measured in pixels, is 0.69. 

### 2.4. Triangulation and 3D Reconstruction

Triangulation [27] is a technique that is used to determine the three-dimensional position of an object or scene from information collected by one or more cameras that capture images and video in two dimensions. Of course, OpenPose has a library for 3D reconstruction, but we did not utilize this library for our reconstruction. Instead, we used OpenPose solely for detecting key points in images. There are a few reasons for this choice. Firstly, OpenPose has limitations regarding the number of people it can detect, allowing only one person at a time; this could be a limitation to the future development of this research. Additionally, the engineers behind OpenPose have stated that they do not actively update the library for 3D reconstruction. For these two main reasons, we opted to implement an algorithm inside MATLAB, and write the code specifically for the 3D reconstruction process. In practice, triangulation uses geometric projection techniques to calculate the location of a given object in space. In fact, when an object is taken from multiple points of view (≥2), each camera projects the image of the object on a different two-dimensional plane. If the camera positions and projected images are known, triangulation can be used to obtain the three-dimensional position of the object. 

**Figure 9:**
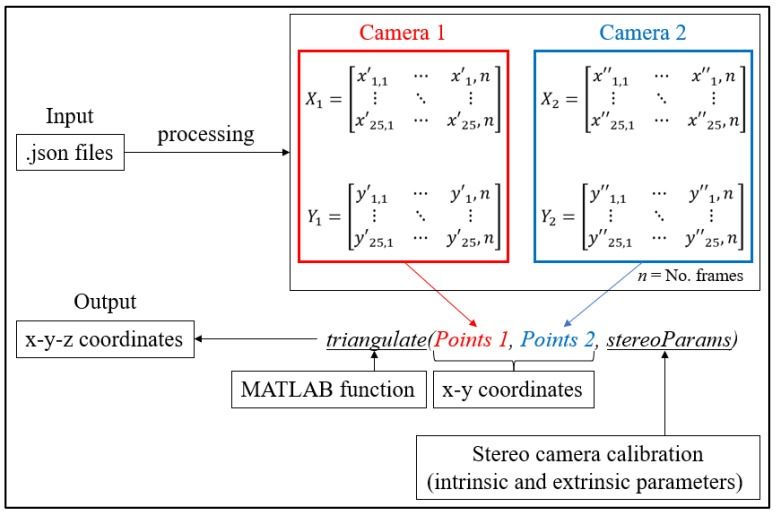
Schematization of the implemented MATLAB script.

Using the coordinates of the key points, and the calibration parameters, the triangulation procedure was carried out, which then allowed the obtention of a representation of the key points of the rider in the three-dimensional space. Figure 9 shows a summary diagram of the MATLAB script used to obtain the 3D reconstruction.

As can be seen in the figure, the implementation of the script in MATLAB starts with .json files from the OpenPose application, in which the 2D coordinates of the detected key points (from each camera) are stored. Through the processing of the .json files, the x–y coordinates for the two recordings are stored separately. For example, X1 is a 25 × n matrix in which all the x coordinates of the 25 key points are stored for each frame (n) of the video. At the end, the application of the “triangulate” MATLAB function allows the obtention of the three coordinates of the key points. In particular, the triangulate function is fed with the two 2D sets of points, and the information from the stereo camera calibration (also performed in MATLAB) containing the intrinsic and extrinsic parameters of the stereo camera. 

The 3D reconstruction time for processing a single frame on an Intel Core i7-9750H @ 2.6 GHz and 16 GB of RAM is 0.17 s. 

## 3. Results

### 3.1. Mannequin Reconstruction

In the previous chapter, the methodology used in this study was discussed, which involved the use of OpenPose on two cameras to capture a rider on a motorbike from different angles.

In order to assess the variability in the results, and to test the method’s reliability, tests were conducted on two different riders, referred to as Subject 1 and Subject 2. It is worth noting that camera calibration was performed only at the beginning of the tests, and it remains valid for both subjects.

**Figure 10:**
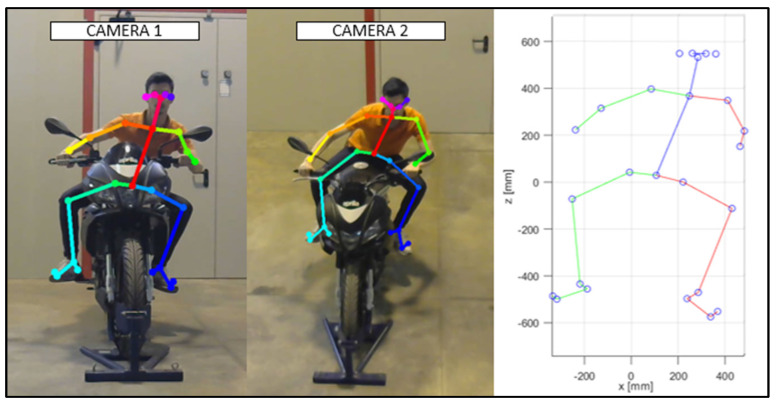
An instance of the OpenPose application, and of the scatter plot of the detected key points (Subject 1).

**Figure 11:**
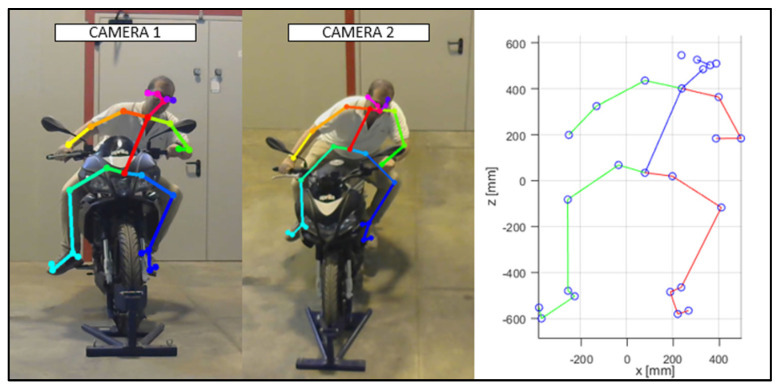
An instance of the OpenPose application, and of the scatter plot of the detected key points (Subject 2).

As discussed in the previous chapter, the JSON files, obtained via OpenPose from the videos recorded by the cameras (Figure 10 and Figure 11), were imported into the MATLAB script, with the parameters of the stereo calibration, to reconstruct the 3D mannequin. As can be seen in Figure 10 and Figure 11, the subject riding the motorcycle, from the point of view of both cameras, is shown. It is possible to see the OpenPose application that reconstructs the 2D mannequin through the identification of the key points. On the right side of the same image, an example of the front view (x–z plane) of the 3D reconstruction is shown. The colors chosen, red and green, for the upper and lower limbs, refer, respectively, to the right and left sides of the body.

The reconstructed mannequin can be plotted frame by frame, thus providing the possibility of monitoring the movement of the studied person. The mannequin is articulated at several joints, which are identified via the joining of the corresponding key points. 

**Figure 12:**
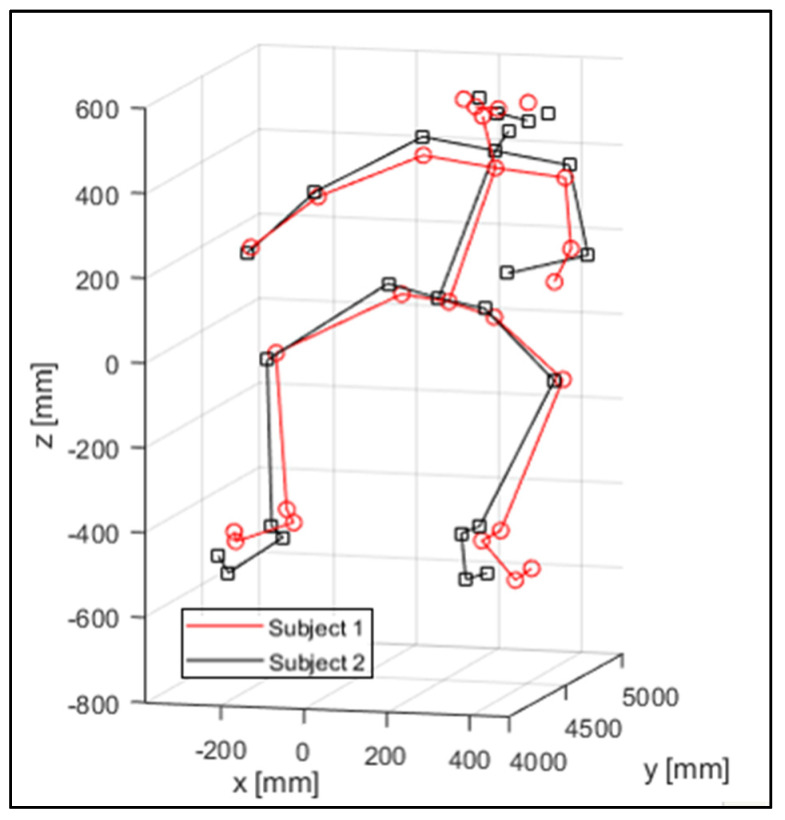
3D key-point reconstruction.

Figure 12 shows a spatial view of the 3D reconstruction of the mannequins of the two subjects in an instantaneous, similar position, assumed while riding the motorcycle. 

**Figure 13:**
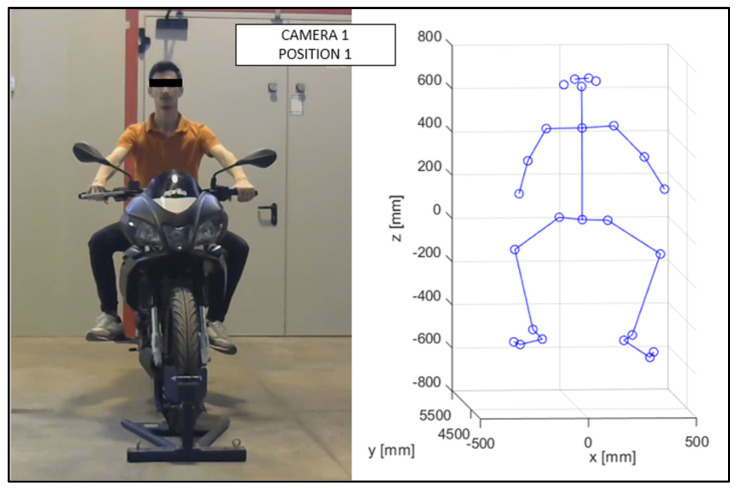
Reconstruction of Position 1 for Subject 1.

**Figure 14:**
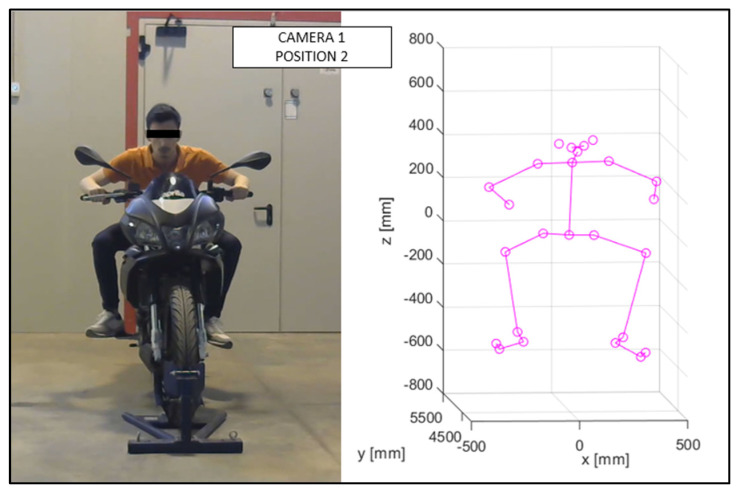
Reconstruction of Position 2 for Subject 1.

**Figure 15:**
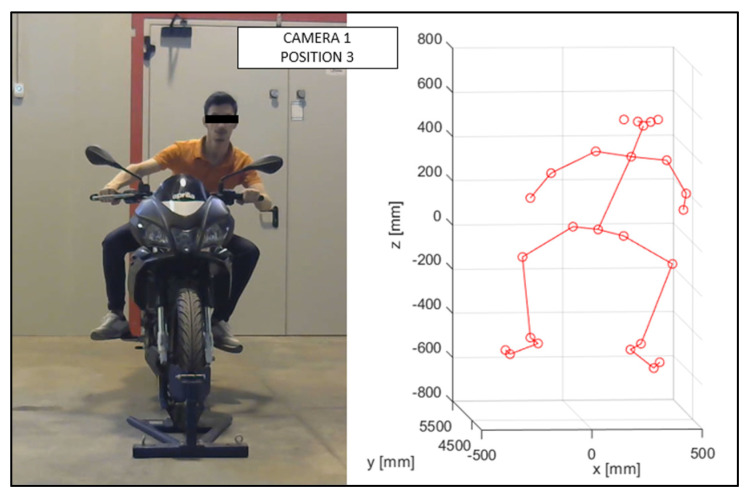
Reconstruction of Position 3 for Subject 1.

**Figure 16:**
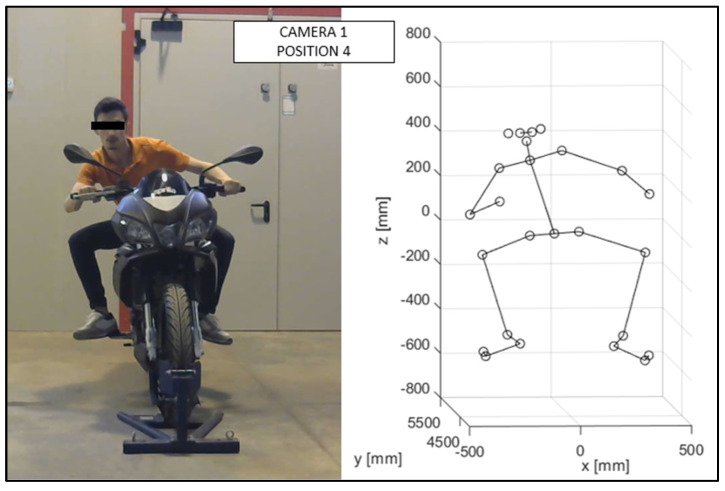
Reconstruction of Position 4 for Subject 1.

**Figure 17:**
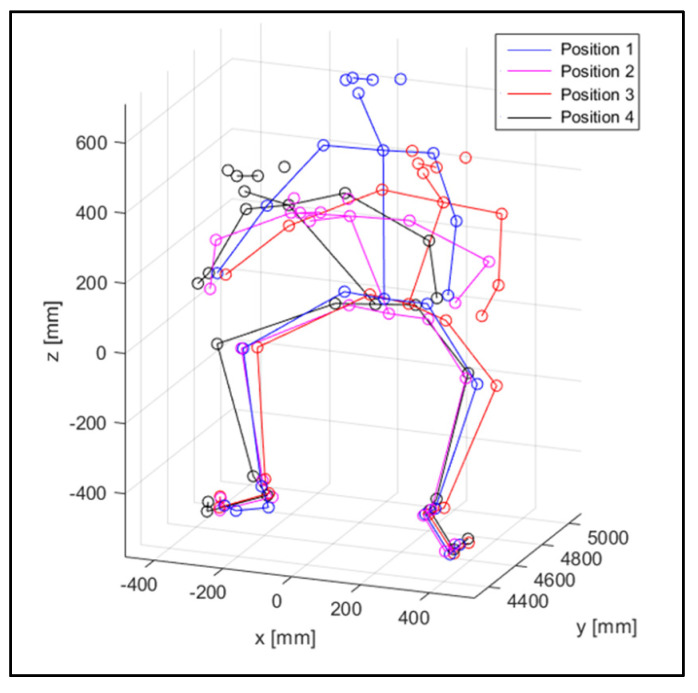
Comparison of the four positions for Subject 1.

**Figure 18:**
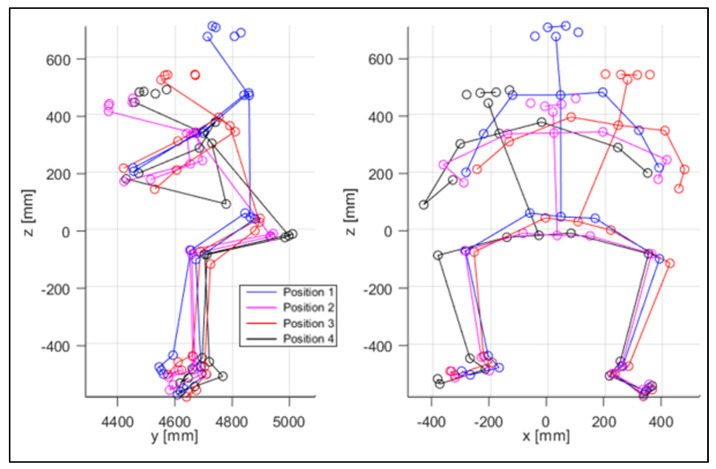
Comparison of the four positions, from the side and from the front view, for Subject 1.

The virtual mannequin can be displayed at various time steps, offering the possibility of comparing two or more positions assumed during the ride. Figure 13, Figure 14, Figure 15, Figure 16, Figure 17 and Figure 18 show four postures performed by Subject 1 that are typically assumed by the human body on the motorcycle while riding. The images and, in particular, the 3D reconstructions, highlight the capacity of the algorithm to detect all the postures assumed by the rider. The frames on the left are extracted from the recording of Camera 1, and are reported only as a reference for a better understanding of the 3D reconstruction. The chosen postures are the ones typically assumed during a ride, and they are listed in Table 2 for Subject 1, and Table 3 for Subject 2 (for every position, the time in which the posture is reached during the acquisition is reported). Figure 17 and Figure 18 show a comparison of the four 3D-reconstructed positions assumed by Subject 1 in the same plot. 

The lengths of arms and legs were assessed and analyzed, and their measurements are presented in Table 4.

### 3.2. Distances

**Figure 19:**
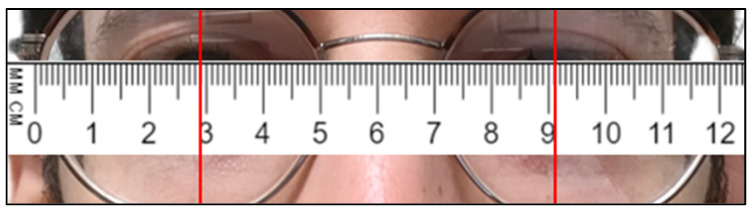
Distance between the eyes’ pupils, measured on Subject 1.

**Figure 20:**
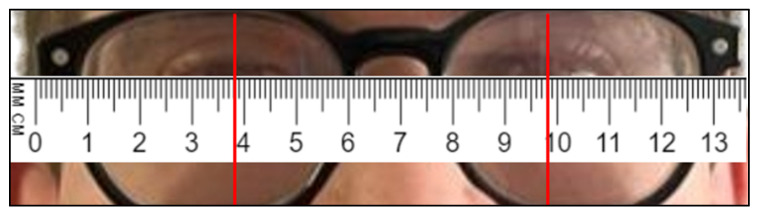
Distance between the eyes’ pupils, measured on Subject 2.

**Figure 21:**
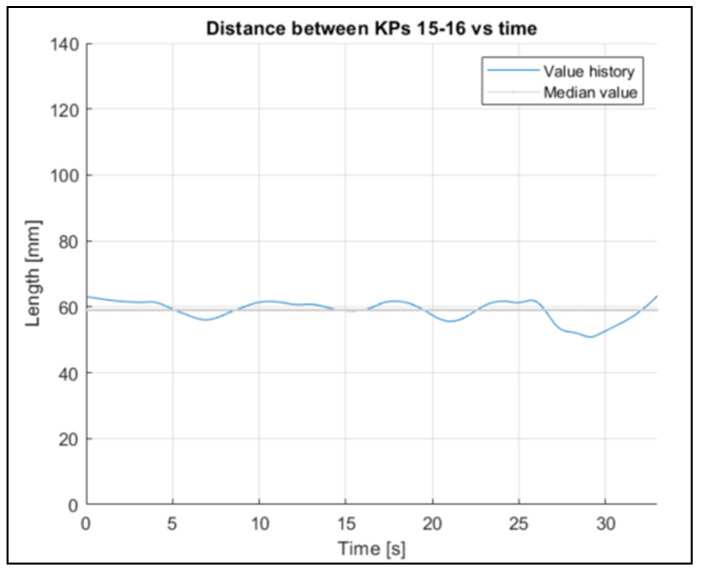
Distance between the eyes during acquisition, for Subject 1.

To verify whether the 3D reconstruction was in accordance with the reality, the trend over time of the distance between the eyes was analyzed. In a preliminary phase, the distance between the pupils of the two riders was recorded: this distance is 62 mm for Subject 1 (Figure 19), and 60 mm for Subject 2 (Figure 20). Distance between the eyes during acquisition, for Subject 1, is shown in Figure 21.

As reported in Table 5 and Table 6, the mean values recorded during the acquisition via OpenPose are 58 mm and 65 mm, respectively. The measures captured via OpenPose show a fluctuation around the mean value, and this can be caused by many factors: a change in light, a change in position, or small differences in the OpenPose definition of the key points. However, the recorded mean value is close to the value measured on the rider during the experimental phase. 

**Figure 22:**
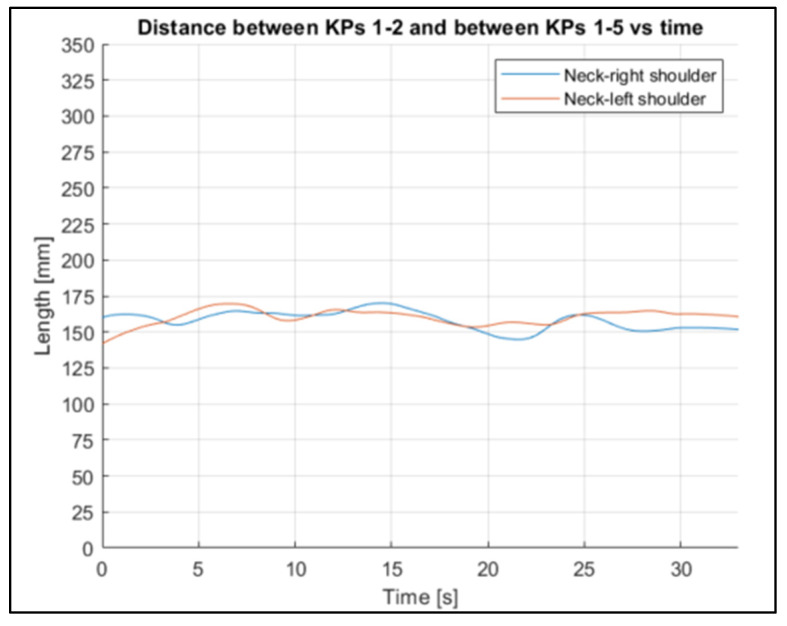
Neck–shoulder joint length trends for Subject 1.

**Figure 23:**
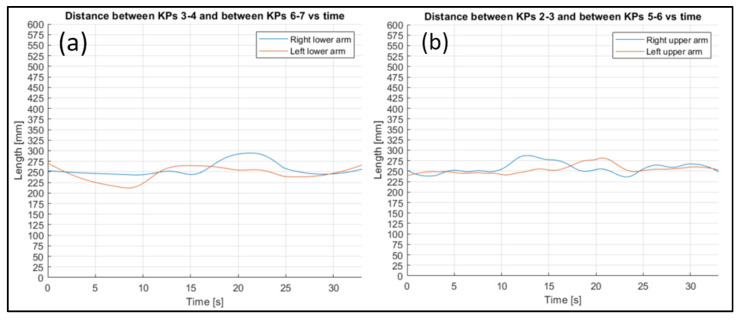
Comparison between lower (**a**) and upper (**b**) arm length trends for Subject 1.

**Figure 24:**
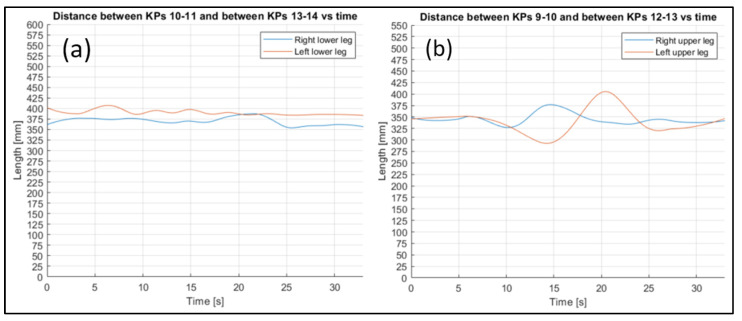
Comparison between the lower (**a**) and upper (**b**) leg length trends for Subject 1.

To evaluate the stability of the reconstruction process, the fluctuation in some other joints’ lengths were evaluated (Figure 22, Figure 23 and Figure 24 refer to Subject 1).

The fluctuations over time in all the lengths are small, considering the actual lengths of the corresponding body parts.

The mean lengths of the joints of Subject 1, and the differences between the left and right side are reported in Table 4, while Table 5 compares the actual lengths of the body segment, and the length measured in the 3D reconstruction. The same information about Subject 2 is reported in Table 6 and Table 7.

**Figure 25:**
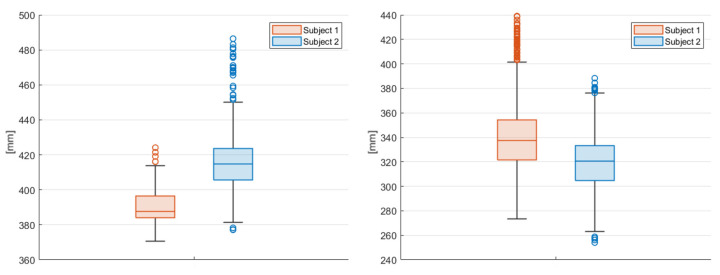
Box plot of the left lower leg (**left**) and of the left upper leg (**right**) of the two subjects. On the y-axis is the length of the joint in mm.

**Figure 26:**
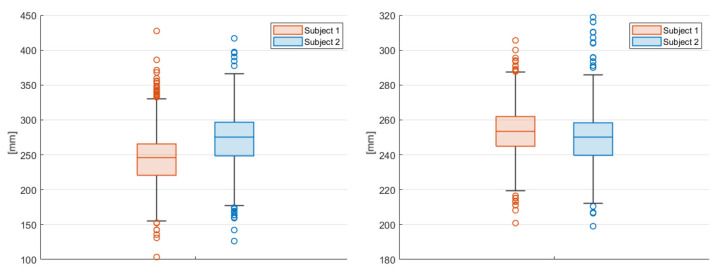
Box plot of the left lower arm (**left**), and of the left upper arm (**right**) of the two subjects. On the y-axis is the length of the joint in mm.

As shown in the tables above, the standard deviations of the two subjects are similar in all the lengths tested. The measure with the highest difference in terms of standard deviation is the lower arm; in this case the standard deviation for Subject 1 is 43, and for Subject 2 is 29. Figure 25 and Figure 26 show the box plots of the lengths of the left legs (lower and upper) and the lengths of the left arms (lower and upper). The box plots highlight a similar distribution for the two subjects, except for the left lower leg, where Subject 1 shows an asymmetric behavior. The right parts of the legs and arms show a similar trend, and they are not plotted.

### 3.3. Angles

**Figure 27:**
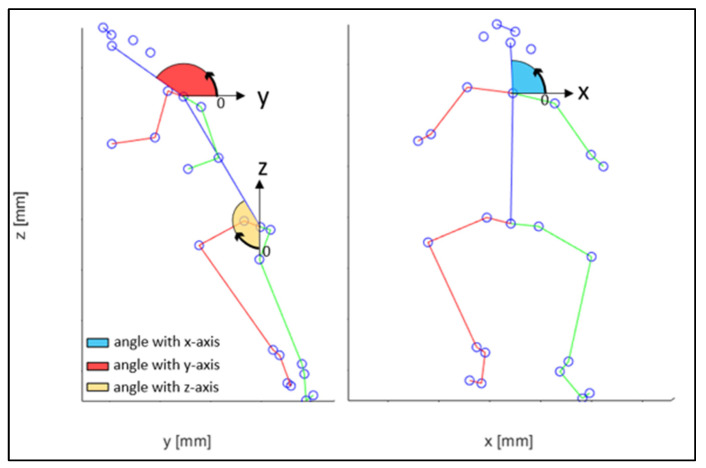
Coordinate system reference.

The angles formed by the main joints and the coordinate axis (Figure 27) are recorded over time. 

**Figure 28:**
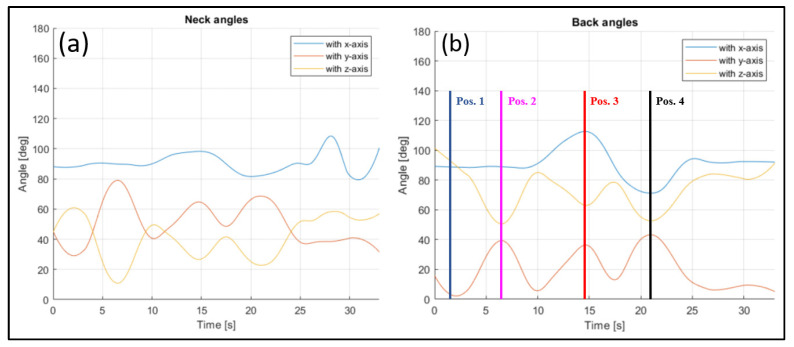
Angles of the neck (**a**), and the back (**b**) over time (Subject 1).

**Figure 29:**
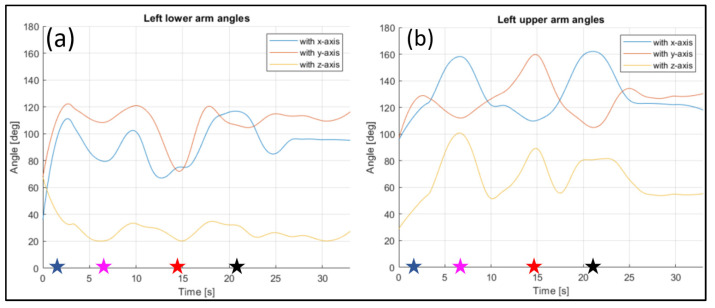
Angles of the left lower arm (**a**), and the left upper arm (**b**) over time (Subject 1). The star-shaped markers indicate the beginning of different positions at various time points (Table 2), and the colors are those used in Figure 28b.

**Figure 30:**
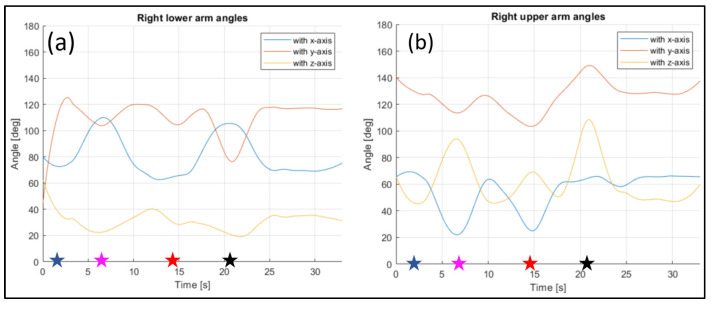
Angles of the right lower arm (**a**), and the right upper arm (**b**) over time (Subject 1). The star-shaped markers indicate the beginning of different positions at various time points (Table 2), and the colors are those used in Figure 28b.

**Figure 31:**
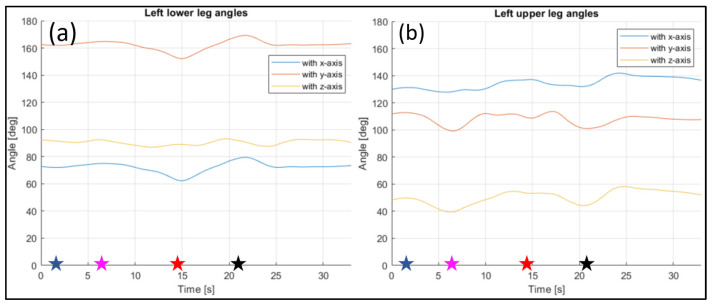
Angles of the left lower leg (**a**), and the left upper leg (**b**) over time (Subject 1). The star-shaped markers indicate the beginning of different positions at various time points (Table 2), and the colors are those used in Figure 28b.

**Figure 32:**
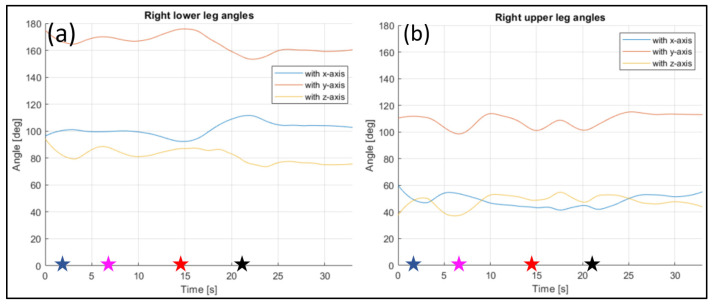
Angles of the right lower leg (**a**) and the right upper leg (**b**) over time (Subject 1). The star-shaped markers indicate the beginning of different positions at various time points (Table 2), and the colors are those used in Figure 28b.

The evaluated joints are the one between the nose and neck, to obtain the angle of the head, the back (defined between the neck and the mid hip), the upper arms (between the shoulder and elbow), the lower arms (between the elbow and wrist), the upper legs (between the hip and knee) and the lower legs (between the knee and ankle). Figure 28, Figure 29, Figure 30, Figure 31 and Figure 32 refer to Subject 1.

For instance, the joint between the nose and the neck, which gives the orientation of the head, can be studied. The angle is measured as in Figure 28, and it is higher when the rider is bent on the fuel tank, and becomes lower when they straighten up.

In Figure 28, the angle formed by the back with the y-axis is tracked, too. This is the angle that most influences the sense of comfort of the rider.

In Figure 29 and Figure 30, the angles of the left arm and the right arm are represented. The arms are separated in two different segments: the upper arm, from the shoulder to the elbow, and the lower arm, from the elbow to the wrist.

The angles of the legs are reported in Figure 31 and Figure 32. The upper leg is defined as stretching between the hip and the knee, while the lower leg is between the knee and the ankle.

**Figure 33:**
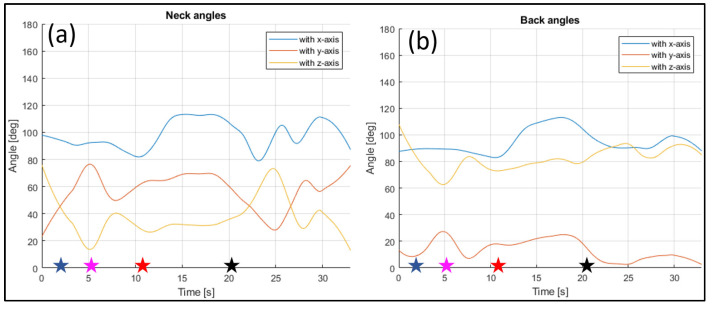
Angles of the neck (**a**), and the back (**b**) over time (Subject 2). The star-shaped markers indicate the beginning of different positions at various time points (Table 3), and the colors are those used in Figure 28b.

**Figure 34:**
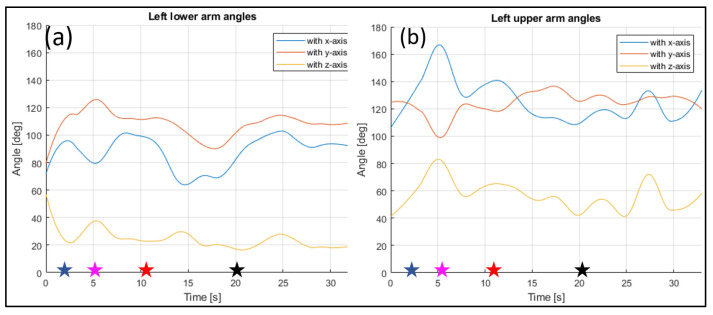
Angles of the left lower arm (**a**), and the left upper arm (**b**) over time (Subject 2. The star-shaped markers indicate the beginning of different positions at various time points (Table 3), and the colors are those used in Figure 28b.

**Figure 35:**
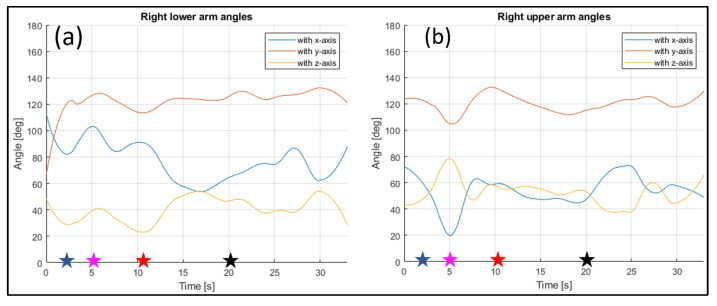
Angles of the right lower arm (**a**), and the right upper arm (**b**) over time (Subject 2). The star-shaped markers indicate the beginning of different positions at various time points (Table 3), and the colors are those used in Figure 28b.

**Figure 36:**
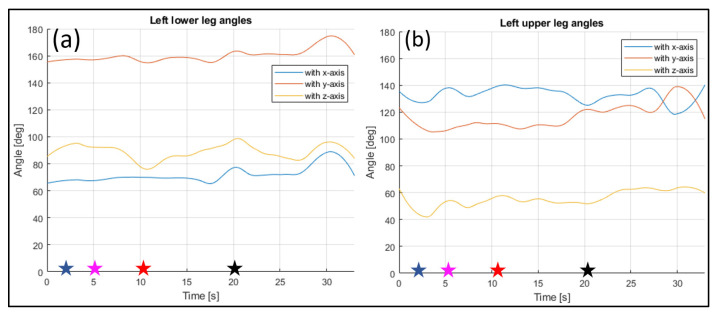
Angles of the left lower leg (**a**), and the left upper leg (**b**) over time (Subject 2. The star-shaped markers indicate the beginning of different positions at various time points (Table 3), and the colors are those used in Figure 28b.

**Figure 37:**
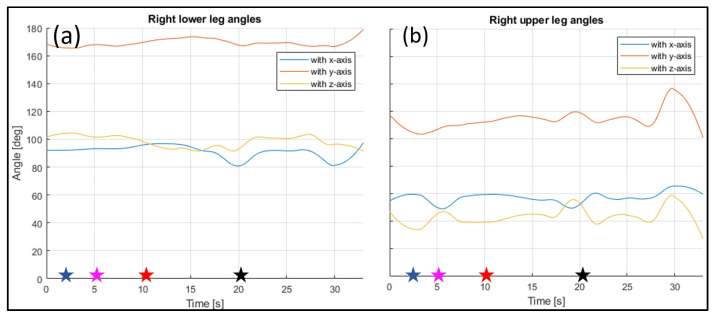
Angles of the right lower leg (**a**), and the right upper leg (**b**) over time (Subject 2. The star-shaped markers indicate the beginning of different positions at various time points (Table 3), and the colors are those used in Figure 28b.

The same information were gathered for Subject 2 in similar plot from Figure 33, Figure 34, Figure 35, Figure 36 and Figure 37.

## 4. Discussion

Thanks to the registration of each frame, it is possible to analyze each posture, and the evolution of the posture between different positions. For instance, in Figure 28, it is possible to see how the back angle changes clearly during the movement between the positions assumed and listed in Table 2. When the rider is in Position 1, the angle formed by the back and x-axis is almost 90 degrees, and it remains constant when the rider lowers on the fuel tank, to assume Position 2. In Position 3, a maximum of the angle is registered, with the rider performing a left turn; in Position 4, the value of the angle reaches an almost symmetrical minimum when a right turn is performed. The angle between the back and the y-axis is zero when the rider is straight (Position 1), then it increases as the rider lowers on the fuel tank, until Position 2 is reached. The angle increases again when the rider lowers in Positions 3 and 4 to perform the turns. The angle between the back and the z-axis shows the opposite behavior to the previously described angle, with it being defined as the complementary angle at 90 degrees to the angle with the y-axis.

The position of the wrists and feet remains practically unchanged as, in both postures, they remain anchored, respectively, to the grips and to the pedals. The authors consider this invariance a strong point of the proposed method, as it guarantees its reproducibility.

Figure 21 shows that the distance between the eyes shows a minimum variation during the acquisition, with a mean value of 58 mm. The real distance measured on the rider is approximately 62 mm, so the reconstructed value can be considered reliable.

Through the three-dimensional reconstruction, it was possible to trace both the distances between the various points that represent the various parts of the body mentioned above, and the angles that these segments assume with respect to the axes. Figure 28, Figure 29, Figure 30, Figure 31 and Figure 32 show the variation in the angles formed by the main joints with the coordinate axis. The trends in some characteristic distances are also shown (from Figure 22, Figure 23 and Figure 24). These shown trends are the distances between two key points which lie on points of the human body rigidly connected to each other, or which admit negligible relative movements, i.e., KPs 1–2, which represent the distance between the neck and the right shoulder (Figure 20). In Figure 20, it is possible to see how the lengths of both the segments (right and left) remain between 145 mm and 175 mm, with average values of 160 mm for the left segment, and 158 mm for the right one.

Through analyzing the other distances between key points related to rigidly connected parts of the body (such as the arms and legs), the following can be observed. By plotting the distance trends, and highlighting the difference between the right and left limbs, it is possible to see that, although there are some fluctuations, the average value remains almost the same.

Table 4 shows the mean values of the lengths of the rigidly connected parts of the body, and the percentage difference between the right and left side. In Table 5, the mean values of the same parts are shown, and compared with the actual lengths, also in percentage terms, obtained through taking real measurements on the rider themself.

As can be seen from the data listed in Table 5, the reconstructed lengths show a low error for both the arms and the legs.

Similar reasoning to that performed for Subject 1 can also be applied to Subject 2, relying on Table 6 and Table 7, and Figure 33, Figure 34, Figure 35, Figure 36 and Figure 37.

Further development of the calibration process can be implemented in future works, to decrease the difference between the real and reconstructed lengths. However, it is important to say that this mismatch between the real and virtual measurements does not impact the ergonomic interest of the work, because the angles between limbs are not length-dependent.

## 5. Conclusions

In this study, a script was developed to reconstruct a human in 3D, to detect posture changes over time. The method relied on tracking the lengths and angles of joints, obtained via OpenPose. It was found that the lengths of the joints corresponding to a particular body part were relatively constant during both acquisition and reconstruction, which is consistent with what would be expected anatomically.

This finding suggests that the method is reliable for detecting posture changes over time, as changes in joint length could potentially interfere with accurate tracking. Additionally, the results suggest that the 3D reconstruction algorithm is robust for tracking human joints across time, as it consistently detects the same joint lengths over multiple frames.

Future research could involve the realization of an algorithm that, using as its input the 3D point dataset, generates a digital twin model on any computer-aided design or computer-aided engineering software with an automated process. 

This model, which would have to replicate the same movements as the real subject in real time, could be useful in performing interactive ergonomics analysis.

The digital twin could be also integrated into software such as OpenSim, to simulate and analyze the dynamic forces acting on the human body for the recorded movements. 

A digital twin model could be also useful in performing dynamic computer-aided engineering simulations, such as computational fluid dynamics simulations, to investigate the impact of a movement of the body on the aerodynamic forces and moments of the motorcycle–rider system. This information can be used to extract the best aerodynamic position to be replicated in wind tunnel testing, or on the track in motorsport applications. However, there are some limitations to the study that should be considered. Firstly, the sample size was relatively small, consisting of only one participant. Future research could explore the validity of the method across a larger sample size. Additionally, the study only focused on detecting posture changes during controlled movements in a laboratory setting. It remains unclear whether the method would be equally effective in detecting natural posture changes in real-world settings. The main outcome of the paper is to provide the expertise required to implement alternative methods to those currently used in the motorcycle industry for postural evaluation—specifically, the use and configuration of marker-less tools for 3D reconstruction that can elevate the current standards, based on the use of 2D images, to a higher and more detailed level of motorcycle posture assessment.

## Figures and Tables

**Table 1:** The technical features of the two cameras.

Feature	Value
Brand	Logitech
Model	270C
Video format	HD 720p
Frame rate	30 fps
Field of view (FoV)	60°
Auto focus	No

**Table 2:** Assumed positions during the recording for Subject 1.

Positions		Time [s]
Position 1	Straight	1.5
Position 2	On the tank	6.5
Position 3	Left turn	14.5
Position 4	Right turn	21.4

**Table 3:** Assumed positions during the recording for Subject 2.

Positions		Time [s]
Position 1B	Straight	2.3
Position 2B	On the tank	5.5
Position 3B	Right turn	11.2
Position 4B	Left turn	20.5

**Table 4:** The mean (μ) and standard deviation (σ) lengths of the reconstructed arms and legs (Subject 1).

Joint	Left-Side Length μ (σ) [mm]	Right-Side Length μ (σ) [mm]	Right-to-Left Difference [%]
Upper arm	254 (14)	257 (17)	+1.2
Lower arm	245 (39)	256 (47)	+4.3
Upper leg	340 (31)	344 (21)	+1.2
Lower leg	390 (9)	370 (10)	−5.4

**Table 5:** Comparison between reconstructed and real lengths (Subject 1).

Joint	Real Length [mm]	Reconstructed Length μ (σ) [mm]	Reconstructed-to-Real Difference [%]
Distance between eyes	62	58 (6)	−6.9
Upper arm	275	255 (16)	−7.8
Lower arm	222	250 (43)	+11.2
Upper leg	373	342 (26)	−9.0
Lower leg	380	380 (14)	+9.7

**Table 6:** Comparison between the reconstructed and real lengths (Subject 2).

Joint	Real Length [mm]	Reconstructed Length μ (σ) [mm]	Reconstructed-to-Real Difference [%]
Distance between eyes	60	65 (8)	+7.7
Upper arm	290	253 (20)	−14.6
Lower arm	270	255 (29)	−5.8
Upper leg	390	335 (26)	−16.4
Lower leg	400	410 (17)	+2.5

**Table 7:** The mean (μ) and standard deviation (σ) lengths of the reconstructed arms and legs (Subject 2).

Joint	Left-Side Length μ (σ) [mm]	Right-Side Length μ (σ) [mm]	Right-to-Left Difference [%]
Upper arm	249 (16)	257 (23)	+3.1
Lower arm	270 (38)	240 (20)	−12.5
Upper leg	320 (22)	350 (30)	+8.5
Lower leg	415 (15)	406 (19)	−2.2

## Data Availability

Data is available upon request.
